# Application of Telemedicine for Preliminary Screening of Autism Spectrum Disorder

**DOI:** 10.3389/fped.2021.745597

**Published:** 2022-01-18

**Authors:** Ting Qiu, Heng Zhang, Conghua Zhou, Qilong Tang, Lizhen Wang, Xiaoyan Ke

**Affiliations:** ^1^Departments of Child Health Care, Wuxi Maternity and Child Health Care Hospital, Nanjing Medical University, Wuxi, China; ^2^School of Computer Science and Telecommunication Engineering, Jiangsu University, Zhenjiang, China; ^3^Child Mental Health Research Center, Nanjing Brain Hospital, Nanjing Medical University, Nanjing, China

**Keywords:** autism spectrum disorder, telemedicine, preliminary screening, WeChat, CHAT-23

## Abstract

**Objective:**

Preliminary screening for autism spectrum disorder (ASD) is mainly performed offline in China. This method is time consuming, labor intensive, inefficient and costly. These complications limit its routine implementation in some hospitals and child health institutions, especially community health service centers. Thus, the present study explored the clinical applicability of an online screening system for ASD detection based on telemedicine technology.

**Methods:**

The online screening system designed based on the WeChat platform and section A of the Chinese-validated version of the checklist for autism in toddlers (CHAT-23-A) and combined with an independent Research and Development (R&D) program. The sensitivity and specificity were 0.92 and 0.90, respectively, and the area under the receiver operating characteristic curve (AUC) values for all 23 items and 7 key items were 0.934 and 0.91, respectively.

**Results:**

The online screening system based on telemedicine technology was not limited by time, space, region, or medical resources and showed high sensitivity, specificity, and diagnostic efficiency for ASD.

**Conclusion:**

The online screening system based on telemedicine technology is suitable for large-scale population ASD screening in childcare institutions.

## Background

Autism spectrum disorder (ASD) is a neurodevelopmental disorder characterized by deficits in social interaction and restricted, repetitive behaviors (RRBs). With refined diagnostic criteria, the public's awareness of ASD has continued to increase. Many countries have reported that the prevalence of ASD is increasing year by year, and the global prevalence rate is estimated to be ~1–2% (1). According to reports, 1 in 54 children in the United States may have ASD (2). To date, only a regional survey of ASD has been conducted in China, and a national epidemiological survey has not yet been available. A meta-analysis based on studies in China revealed that the prevalence of ASD was 39.2/10,000, which was lower than the reported rates by other countries. The study explained that due to the use of varied screening and diagnostic tools, interpretation of the results should be cautious (3). Another study in 2019 demonstrated 1% prevalence of autism in Jilin city in western China (4). A recent multicenter survey conducted in China showed that the prevalence of ASD was 0.7%, but the participants were 6–12 years old, which excluded the younger populations (5).

Currently, there are no specific drugs available for the treatment of ASD. Early diagnosis and intervention are critical to improving prognosis. Previous studies have reported that early intervention can improve the cognitive, language, and adaptive abilities of children with ASD (6). Although ASD can be diagnosed as early as 24 months of age, studies have shown that the average age of diagnosis of children with ASD in low- and middle-income countries varies between 45 and 72 months (7). Studies from high-income countries have shown that the mean age of diagnosis in ASD children is delayed to 50 and 60 months of age (7). Early ASD screening is the first step in early diagnosis and intervention, and the cost-effectiveness of early screening for ASD is better than comprehensive diagnosis without screening (8).

The checklist for autism in toddlers (CHAT) (9), the modified checklist for autism in toddlers (M-CHAT) (10), and the Chinese version of the Checklist for Autism in Toddlers (CHAT-23) (11) are commonly used screening tools in China. The CHAT-23 was the first localized screening tool designed for Chinese ASD children in 2004. In 2010, Wu et al. (12) first introduced this approach in Shanghai and verified the effectiveness of the CHAT-23 as an early screening tool based on its diagnostic sensitivity and specificity (0.941 and 0.884, respectively). Ren et al. (13) reported slightly higher sensitivity and specificity values of 0.971 and 0.961, respectively. The CHAT-23 is a localized ASD screening tool that is being gradually promoted in China. In 2017, the developmental behavior group of the science branch of the Chinese Medical Association formulated an expert consensus on the early identification and screening of ASD for the first time. According to the expert consensus (14), ASD screening for 18- and 24-month-old infants based on the three-level prevention and monitoring network of child healthcare should be a routine item in China, and the CHAT-23-A was recommended as the instrument for preliminary screening. However, at present, ASD screening in China is mainly performed offline. The successful implementation of screening requires specific conditions. First, medical staff and screening subjects need face-to-face interaction. Second, professional screening personnel are required to distribute questionnaires and judge the results, which requires private and quiet places. The high cost of human resources and long screening time limit the implementation of routine large-scale ASD screening in some hospitals, especially community health service centers.

With the continuous development of the internet and the popularization of smartphones in China, relying on internet technology to provide telemedicine and healthcare services has become a reality. Telemedicine can eliminate time, space and geographical restrictions in patient management, and convey information to the doctors thoroughly, accurately and rapidly, thereby establishing a new channel for doctor–patient communication. This field has also become a research hotspot in recent years. WeChat is a mobile application widely used in the medical industry due to its versatility in the management of chronic diseases (15). However, there are no reports on the application of telemedicine for early ASD screening. Therefore, to resolve the disadvantages of offline questionnaire screening, we designed a remote screening system for ASD based on the WeChat platform and CHAT-23-A, combined with independent Research and Development (R&D) procedures. This system was applied to explore its practical clinical applicability in detecting ASD among children aged 18–30 months who visited our child healthcare department.

## Methods

### Participants

All participants were recruited at the child healthcare department of the Affiliated Wuxi Maternity and Child Health Care Hospital of Nanjing Medical University, China, from July 2020 to June 2021. A total of 50 children, aged 18–30 months, were diagnosed with ASD by two licensed and experienced child psychologists based on the Diagnostic and Statistical Manual of Mental Disorders, Fifth Edition (DSM-5) (16) diagnostic criteria and standardized clinical assessments, including the Childhood Autism Rating Scale (CARS) (17). Those with an inconsistent diagnosis were not included in this cohort. Fifty children with typical development were selected as the control group, and sex was matched between the ASD and control group. Subjects with any systemic diseases, history of head injury, genetic syndromes, neurological disorders, or psychiatric illness were excluded from participation. This study was approved by the Academic Ethics Committee of the Affiliated Wuxi Maternity and Child Health Care Hospital of Nanjing Medical University. All guardians of the children agreed to participate and signed an informed consent form. However, they could refuse to participate or withdraw from the study at any time. All data were only used for this study and were kept confidential.

### Screening Tool

The CHAT-23 (11), also termed the “Behavioral and Communication Checklist for Children” in China, was used to screen ASD in children and demonstrated validity and reliability in assessing 18- to 24-month-old children (18). CHAT-23 includes the parent-reported questions as section A (CHAT-23-A) and the observational part as section B (CHAT-23-B).The CHAT-23-A questions are answered by parents, who respond to prompts about the behaviors of children aged 18–24 months with “no,” “occasionally,” “sometimes,” and “often.” According to the positive criteria, ≥6/23 items or ≥2/7 key items (2, 5, 7, 9, 13, 15, and 23) indicate a positive screening.

### Assessments

The CARS was used to assess the degree of the severity of ASD symptoms. The scale consists of 15 items that evaluate interpersonal relationships, imitation, emotional response, physical usability, and correlation with non-living objects. Each item is scored according to severity levels 1–4, and each level has a specific descriptive explanation. A total score of ≤ 30 is considered non-ASD, a total score of 30–35 is considered mild to moderate ASD, and a total score ≥36 is considered severe ASD (19).

Developmental quotient (DQ) is assessed by a specifically trained rater using the neuropsychological examination scale (20, 21) for children aged 0–6 years, which includes five areas: gross motor, fine motor, adaptability, language and social behavior. DQ and mental age are calculated by the following formula: DQ = (mental age/chronological age) ×100; mental age = sum of the scores in the five areas/5, DQ ≥ 85 is assigned as typical (22).

### Online Screening System

The online screening system in this study was developed based on the WeChat platform, CHAT-23-A, and combined with an independent R&D procedure. It was divided into three parts: WeChat public platform, the screening program procedure (independent R&D), and the internal management system (independent R&D, http://gdz.fenghuaxinxi.com/admin/login). First, a WeChat public platform, “the network center for early screening of autism spectrum disorder” was launched and produced a unique QR code. The screening and internal management programs were written and developed by the School of Computer Science of Jiangsu University using Professional Hypertext Preprocessor (PHP) language and PhpStorm development tools. A browser/server (B/S) server structure was used to design the screening procedure by docking different interfaces of the WeChat public platform to achieve different functions. Users can utilize the CHAT-23-A online through its WeChat custom menu interface. The screening results can be sent to the doctor and user through the CHAT-23-A message interface on WeChat. Then, the user and the doctor can communicate via WeChat's customer service interface.

**Figure 1:**
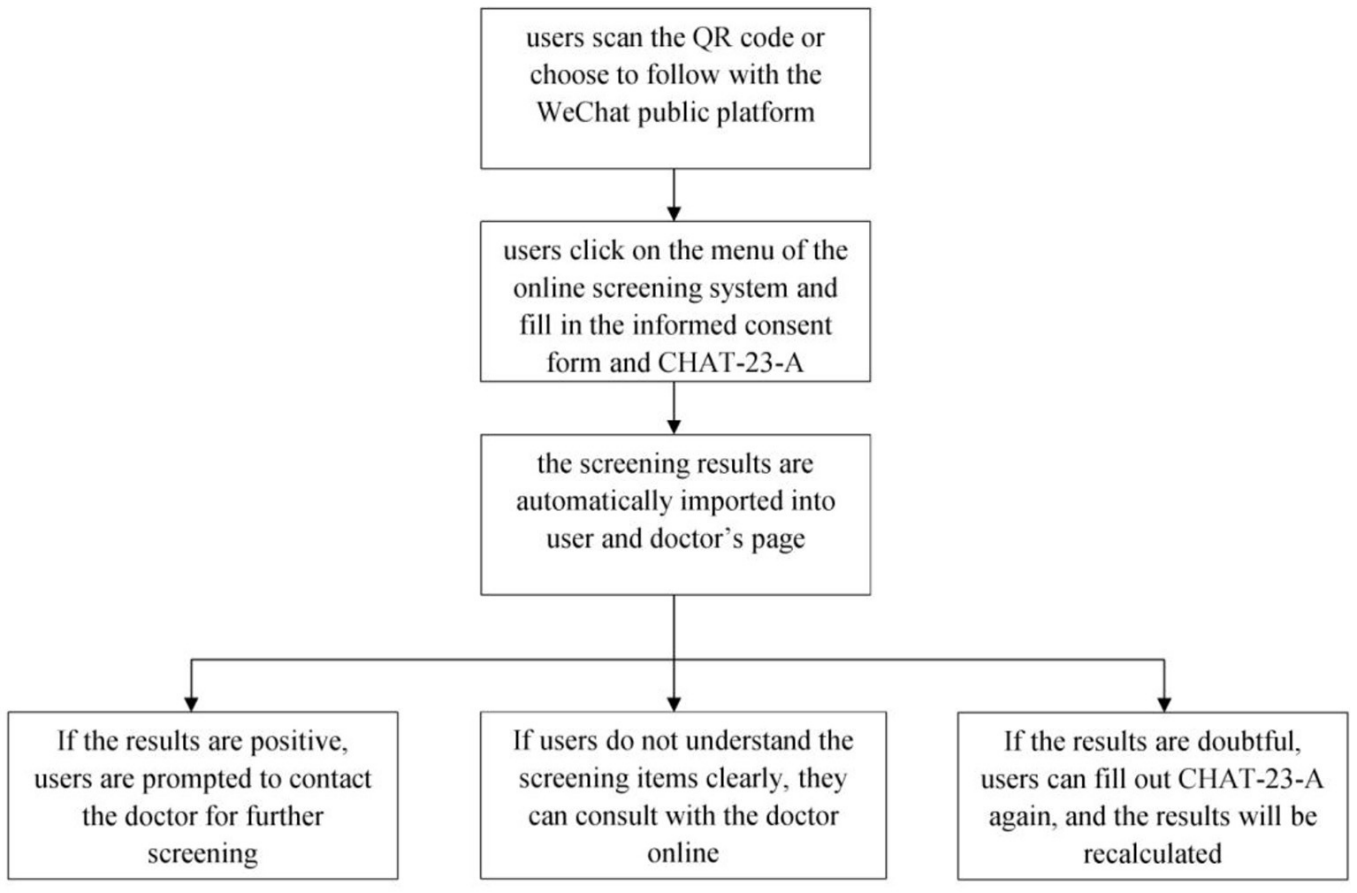
Flow diagram of the online screening system.

In addition, screening data can be displayed electronically through internal management procedures for statistical and management purposes. In summary, the online screening system automatically presents results, sends them to doctors and users, collects data, and provides online consultation (Figure 1).

### Operation Process

The operation process of the online screening system is as follows: users scan the QR code or choose to follow “the network center for early screening of autism spectrum disorder” on the WeChat public platform. Then, the users can go to the WeChat public platform on their smartphones, click on the menu of the online screening system and fill in the informed consent form and the CHAT-23-A questionnaire. Upon completion, the screening results are automatically imported into the internal management system and sent to the platform's user and doctor's pages immediately. If the screening results are positive, the user is prompted to contact the doctor for further screening. If the users do not understand the screening items clearly, they can send messages, pictures, or videos to consult with the doctor online. If the screening results are doubtful, the user can fill out the questionnaire again, and the results will be recalculated and presented.

### Statistical Analysis

SPSS 20.0 statistical software was used for statistical data analysis. The chi-square and *t*-tests were used to determine the differences in sex, age, and DQ between the two groups. The screening evaluation indicators, including sensitivity, specificity, positive predictive value, negative predictive value, and kappa value, were calculated. Total scores for the receiver operator characteristic (ROC) curves for 23 items and seven key items were used to evaluate the efficacy of the CHAT-23-A screening method. On the ROC curve, sensitivity is plotted on the y-axis, and 1-specificity is plotted on the x-axis. The area under the ROC curve (AUC) value of 0.90–1 is considered excellent, while 0.80–0.89 represents good and 0.70–0.79 represents fair (23).

## Results

### Baseline Information

We recruited a total of 100 children, including 50 ASD children and 50 typically developing children, aged 18–30 months. No significant difference in sex was observed between the two groups. In addition, the ASD group had significantly lower DQ scores than the TD group (*P* < 0.001) (see Table 1).

**Table 1:** Demographic characteristics of participants.

	**ASD**	**TD**	***T*(χ^2^)**	***P***
Sex (male:female)	43:7	39:11	1.62	0.20
Age (x ± s)	25.54 ± 3.99	23.28 ± 4.70	2.59	0.01^*^
DQ	98.32 ± 4.61	67.93 ± 9.36	−20.42	<0.001^***^

*TD, typical developing children; DQ, developmental quotient*.

* *P < 0.05*;

*** *P < 0.001*.

### Detailed Report of Screening Statistics

The sensitivity and specificity results of CHAT-23-A are shown in Table 2. According to 6/23 standard items or 2/7 key items, among 50 patients in the ASD group, the screening results were positive in 46 and negative in four patients. Additionally, 45 were negative, and five were positive in 50 normal controls. The results showed that the screening sensitivity was 0.92, and the specificity was 0.90. The positive and negative predictive values were 0.902 and 0.918, respectively, and the kappa value was 0.802.

**Table 2:** Sensitivity and specificity of CHAT-23-A.

	**ASD (*n* = 50)**	**TD (*n* = 50)**	**Sensitivity**	**Specificity**	**Positive predictive value**	**Negative predictive value**	**kappa**
CHAT-23-A	46	5	0.92	0.90	0.902	0.918	0.802
2/7 key items	42	5	0.84	0.90	0.894	0.849	0.740
6/23 items	32	0	0.64	1.00	1.00	0.735	0.640

*ASD, autism spectrum disorder; TD, typical developing children*.

*CHAT-23-A, section A of the Chinese-validated version of the checklist for autism in toddlers*.

### Evaluation of Diagnostic Performance With ROC Curve

**Figure 2:**
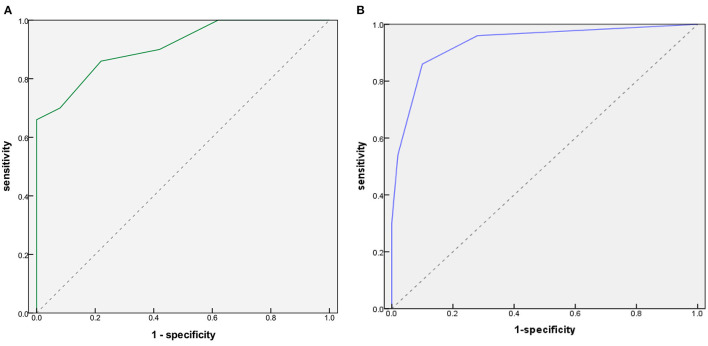
Receiver operating characteristic (ROC) curves for CHAT-23-A. **(A)** Six of 23 items. **(B)** Two of seven key items.

In Figure 2, for all 23 items and seven key items, the AUC estimates were 0.934 (95% CI 0.884–0.983) and 0.91 (95% CI 0.855–0.964), respectively, indicating excellent performance.

## Discussion

In 2009, China launched the basic public health service project (24), which included providing health management services for children aged 0–6 years. All children can receive free physical examinations from birth to 6 years of age (at 1, 3, 6, 8, 12, 18, 24, 36, 48, 60, and 72 months). In 2017, expert consensus in China pointed out that pediatricians in hospitals at all levels should rely on the three-level prevention and monitoring network of child healthcare to routinely screen for ASD in 18- and 24-month-old children (14).

However, with the rapid increase in the prevalence of ASD in recent years, child health institutions are faced with many difficulties in the routine screening for ASD in young children: (1) The number of child health care doctors is limited. Doctors require to conduct many routine health examinations during limited working hours, and no specialized pediatricians or psychologists are responsible for screening. (2) The screening environment is limited. Some community health service centers do not have quiet, private rooms where primary caregivers can fill in CHAT-23-A. (3) The economic feasibility of screening is limited. If routine screening is carried out for all 18- and 24-month-old children in China, many paper-based questionnaires need to be printed, which is very costly and not conducive to environmental protection. (4) The screening efficiency is limited. Offline screening requires medical staff to distribute questionnaires, collect responses and compile statistical results, which is time-consuming and inefficient. (5) The authenticity and screening rate of the questionnaire are limited. In many cases, children are accompanied by grandparents or other non-primary caregivers during their health checkups. Grandparents' comprehension is uneven, as they are not fully aware of their grandchildren's situations, which can lead to an inaccurate CHAT-23-A screening rate. Taken together, the traditional offline screening model requires a large workforce, material resources, time and costs, which results in limited screening rate, feasibility and effectiveness. The above factors are not conducive to the routine screening of ASD in children's health clinics or hospitals at all levels, especially community health service centers.

In China, the WeChat public account platform is widely used in the management and even treatment of chronic diseases (25–27). This study applied telemedicine technology to the early screening of ASD for the first time, used the WeChat public account platform and CHAT-23-A to develop an online screening system named “The Network Center for Early Screening of Autism Spectrum Disorder” and evaluated its clinical applicability. In this study, the diagnostic criteria of DSM-5 combined with the results of CARS evaluation served as the guidelines, and the age of the children for the CHAT-23-A was 30 months, according to the recommendations for China (12). The results showed that the CHAT-23-A fulfilled the standard criteria (6/23 items or 2/7 key items) with a high sensitivity of 0.92, specificity of 0.90, and kappa value of 0.802. The sensitivity was slightly lower than the value of 0.968 reported by Jun et al. (28). A possible reason is that the DQ value of the four children in the ASD group in the study was close to the normal range, so the screening results were negative. Notably, the specificity of the 23 items meeting six of the individual criteria was up to 100%, which was higher than that meeting any standard (99%), according to the study by Jun et al. (28). In addition, the present study used the ROC curve to evaluate the screening value of the scale, and the results proved that it was a comprehensive, accurate, and effective screening test. The data showed that the AUCS of all 23 items and 7 key items were close to one, indicating that the diagnosis was highly authentic. Thus, it can be deduced that the online screening model has high sensitivity and specificity, which satisfies the requirements of high sensitivity and a low missed diagnosis rate of screening tests.

In addition, compared to offline screening, this online screening system could have following advantages. (1) Change in the health care model. This screening is mainly conducted online, allowing children to carry out ASD early screening without leaving their villages or communities and promoting the development of an active child healthcare model that is different from a traditional passive healthcare model. The screening system has broad participation and allows timely interaction of parents, communities, and pediatricians. (2) Automatic reporting of screening results. While primary caregivers fill in children's basic information and screening scale online, the results are synchronized to mobile phones. The online screening system does not need to issue scales, statistics, and analysis results, thus reducing the demand on medical resources, avoiding errors in medical staff judgment, ensuring the accuracy of screening results, and improving screening efficiency. (3) Timely feedback of screening results. Positive screening results will be directly fed back to parents and doctors' mobile phone clients, and a dialog box will pop up to prompt the user to take the children to the community health service center or Wuxi Maternal and Child Health Hospital for further screening. (4) Data management of screening results. The internal management system can automatically aggregate all screening data, including statistics of screening numbers, positive rate, and negative rate in different community health service centers, which facilitate pediatricians to search and manage data. (5) Online consultation with the doctor. The system provides an online consultation service for users that helps parents make convenient further screening appointments. Compared to traditional paper screening methods, the online screening system could have the advantages of high efficiency, accuracy, and cost-effectiveness, which is suitable for early screening in a large sample population.

The online screening system based on telemedicine is not restricted by location, screening frequency, or medical resources, which can greatly reduce the costs for patients and the workload of the medical staff. It can significantly improve efficiency with high sensitivity and specificity, rendering it suitable for clinical ASD screening of infants and young children. Moreover, the online screening system can be used in hospital childcare institutions, especially primary childcare institutions.

However, this study has limitations. First, due to the short application time of the online screening system, its reliability and validity need to be verified with a larger sample. Second, this screening is only performed in ASD children or typically developing children who have been diagnosed. The reports on parents of children who have been diagnosed with ASD may be different than those of parents who do not know much about their children's diagnosis. Therefore, in future research, screening will be tested in general child populations who have not seen a specialist for diagnosis to further explore the screening's performance in detecting new cases of ASD. In addition, nonautistic children with other mental disorders will be added to test the ability of the CHAT-23 to distinguish ASD from other disorders. Furthermore, the advantages of the online screening system compared to traditional paper screening methods mainly come from a functional point of view. In future research, the specific time and cost of the online screening system and traditional offline screening will be calculated statistically. Additionally, it is necessary to strengthen the functional component of the system to serve more families, such as further screening positive cases and developing popular science education related to ASD.

## Data Availability Statement

The raw data supporting the conclusions of this article will be made available by the authors, without undue reservation.

## Ethics Statement

This study was approved by the Academic Ethics Committee of the Affiliated Wuxi Maternity and Child Health Care Hospital of Nanjing Medical University. All guardians of the children agreed to the participation and signed an informed consent form.

## Author Contributions

TQ and XK: designing experiments and guiding supportive contributions. TQ: article writing and statistical analysis of data. HZ and LW: screening and diagnosis of children with autism. CZ and QT: development and maintenance of online screening system. All authors contributed to the article and approved the submitted version.

## Funding

This work was funded by Science and Technology Development Project of Wuxi Science and Technology Bureau (No. WX18IIAN029) and Double Hundred Talent Fund Projects of Wuxi Municipal Health Commission (Nos. HB2020072 and BJ2020082).

## Conflict of Interest

The authors declare that the research was conducted in the absence of any commercial or financial relationships that could be construed as a potential conflict of interest.

## Publisher's Note

All claims expressed in this article are solely those of the authors and do not necessarily represent those of their affiliated organizations, or those of the publisher, the editors and the reviewers. Any product that may be evaluated in this article, or claim that may be made by its manufacturer, is not guaranteed or endorsed by the publisher.
